# Multi-Modal Fusion Frameworks of Subgraph-Optimized Graph Autoencoder for Molecular Property Prediction

**DOI:** 10.1021/acs.jcim.5c02536

**Published:** 2026-01-28

**Authors:** Kaiyuan Zhang, Congyu Han, Fenghua Zhang, Cheng Lin, Quanlong Li, Tianyi Zang, Yanli Zhao

**Affiliations:** † Faculty of Computing, 47822 Harbin Institute of Technology, Harbin, Heilongjiang 150001, China; ‡ National Key Laboratory of Science and Technology on Advanced Composites in Special Environments, Harbin Institute of Technology, Harbin, Heilongjiang 150001, China; § Medical College, 207475 Qinghai University, Xining, Qinghai 810016, China

## Abstract

Molecular property prediction refers to predicting the properties of a given molecular representation. This task is of great significance in fields such as drug design and has garnered widespread attention from researchers. For molecular property prediction, the quality of feature learning plays a decisive role in model performance. Although existing molecular graph models can extract effective feature representations from graph structures, how to better utilize these features across different learning tasks remains an important challenge. This paper proposes a subgraph-optimized Graph Autoencoder (TurboGAE) and several multimodal fusion strategies. By introducing a subgraph-level graph tokenizer, TurboGAE more effectively captures the impact of substructure features (within molecular structures) on molecular properties. For cross-modal molecular features, a rational and effective multimodal feature fusion strategy can align intermodal features during the pretraining phase, leveraging the unique strengths of each modality. The proposed methods demonstrate excellent performance in experiments on downstream tasks.

## Graphical abstract

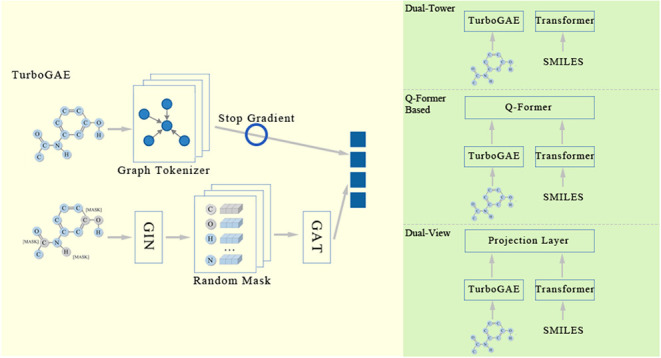

## Introduction

In the 21st century, as global attention to the healthcare sector grows,^1^ technological breakthroughs are transforming the landscape of this industry. Particularly in drug molecule research, the physicochemical properties of these molecules directly determine their performance in practical applications, thereby influencing the innovation and development of the entire industry. For drug molecules, the ability to efficiently and accurately predict properties such as bioactivity, toxicity, and metabolic pathways is crucial significance for accelerating the discovery and clinical application of new drugs.^2^ Therefore, the rapid and accurate capture and prediction of these properties have become one of the core challenges in scientific research.

With the rapid advancement of information technology—particularly breakthroughs in deep learning within the field of artificial intelligence—traditional scientific research methods are gradually being replaced by emerging computer technologies.^3^ As an important branch of artificial intelligence, deep learning has achieved remarkable success in fields including image recognition, speech processing, and natural language understanding. These technologies have not only changed the way we interact with data, but also provided unprecedented support and possibilities for interdisciplinary research.^4^ In recent years, the application of deep learning technologies in the field of drug research and development has increased gradually, showing great potential and advantages, and has become a powerful driving force for innovation and development in these fields.

However, in the specific task of molecular property prediction, existing deep learning methods still have many limitations. First, most methods adopt relatively simple deep learning models, which cannot fully capture the complex structures and property information on molecules. Second, due to the vast number of molecular types and the huge amount of property data, apart from complex large molecules and molecules combined to form compounds, the feasible chemical space of commonly used small molecules has already far exceeded the level that can be determined by experiments. Obtaining these data through traditional density functional theory calculations^5^ or actual experimental determination is not only time-consuming and labor-intensive, but also costly. This has led to a lack of large-scale labeled data sets for molecular property prediction,^6^ which poses many challenges for the training and application of supervised machine learning models.

In the task of molecular property prediction, the quality of feature learning plays a decisive role in the performance of the model. Although existing molecular graph models have been able to extract effective feature representations from the graph structure, how to better utilize these features in different learning tasks remains an important challenge. In our study, we propose TurboGAE, an subgraph-optimized graph autoencoder, and a multimodal framework that includes several fusion strategies. Our main contributions are as follows:

- Simplified Graph Tokenizer: Unlike generic GNN encoders, our tokenizer is explicitly designed to identify and preserve chemical substructures (root functional groups and rings) without learnable parameters. This injects chemical domain knowledge directly into the tokenization process, distinct from purely topological approaches.
- Multi-View Masking: Standard GAEs use a deterministic reconstruction objective. Our Multi-View Masking introduces a probabilistic objective where the model acts as a consistency learner across perturbed views. This mathematically aligns with maximizing the mutual information lower bound.
- Dual-View Consistency: Our fusion strategy achieves alignment between 2D graph and 1D sequence modalities without the need for negative sampling, avoiding the ″false negative″ problem inherent in standard contrastive learning (e.g., GraphCL). We have completely rewritten the Introduction and Contributions sections to rigorously articulate these theoretical differentiators.

## Related Works

With the increasing depth of deep learning applications, the application of deep learning models in interdisciplinary fields has attracted widespread attention from researchers. Recurrent Neural Networks (RNN) and Long Short-Term Memory (LSTM) networks can learn molecular representations through sequence learning. Hou et al. improved long short-term memory (LSTM) network using Bayesian optimization and spatial attention networks. Li et al. proposed a hybrid architecture that combines stacked convolution neural network (CNN) and recurrent neural networks (RNN) layers for representation extraction.^7^ As Transformer and BERT language models have achieved breakthroughs in natural language processing, representing SMILES sequences from a natural language processing perspective using self-supervised models as backbones has shown promising results. Wang et al.^8^ addressed the issue of limited labeled data by pretraining on BERT with Masked Language Modeling (MLM). Fabian et al.^9^ performed pretraining on large-scale unlabeled data using MLM, SMILES equivalence (SMILES-EQ), and molecular physical property prediction tasks (PHYSCHEMPRED). Honda et al.^10^ applied Transformer-based intermediate representations as molecular fingerprints for downstream tasks. With the widespread application of Large Language Models (LLM), how to leverage LLMs for molecular representation has garnered significant attention. Edwards et al.^11^ connected textual and molecular representations by pretraining on the T5 model using molecular representations and textual descriptions.

Representing molecules using graph-structured data is the most natural way to depict them. With the increasing application of GNNs and graph convolution networks (GCNs) in various fields, Kearnes et al.^12^ demonstrated the superiority of GNNs in molecular property prediction. Contrastive self-supervised learning mainly distinguishes between positive and negative samples through data augmentation to learn feature representations, such as MoCo^13^ and SimCLR.^14^ Generative self-supervised learning relies on generative models to understand and model the distribution of data. Sun et al. proposed the InfoGraph^15^ model, which maximizes the mutual information between the entire graph representation and substructure representations at different granularities. You et al.^16^ performed graph-level contrastive learning using a combination of multiple graph augmentations, including node dropping, edge perturbation, subgraph cropping, and feature masking. Hu et al. proposed AttrMask,^17^ and the pret-raining strategy in AttrMask has been widely applied as an important subtask in graph neural network pretraining. Building on this, Hou proposed GraphMAE,^18^ which outperformed graph contrastive learning in downstream tasks by using a remask strategy and a GNN as the decoder. GraphMAE2 ^19^ further enhanced the decoder’s reconstruction capabilities by introducing multiview error. Xia et al. proposed Mole-BERT,^20^ which integrates contrastive learning and graph mask architectures and improves model performance through more complex pretraining tasks. Lin et al.^21^ proposed a self-supervised learning framework that utilizes graph matching and multiple convolution techniques to capture the correspondences between different graphs or subgraphs and deeply fuse the structural and semantic features of molecules. Yoo et al.^22^ proposed HierMolMoE, which achieves more accurate and robust predictions of molecular properties by simultaneously integrating structural features at the atomic, motif, and global levels. Song et al.^23^ proposed the Local Geometry Guided Graph Attention Network (LGGA), which enhances the model’s ability to capture complex local details by integrating local geometric information into the attention and message passing mechanisms of graph neural networks. Liu et al.^24^ incorporated 3D geometric information into the pretraining stage based on molecular graphs, achieving better results. Zeng et al.^25^ used a Transformer as the backbone and introduced a pretraining strategy based on molecular matching. Zhu et al.^26^ used molecular graph prediction of molecular 3D structures and vice versa as pretraining tasks. DMP^27^ performed pretraining based on molecular sequences and molecular graphs, fusing molecular sequence inputs with two-dimensional molecular graph features. Liu et al.^28^ proposed MolCA, which enables LLMs to accept graph-level features as inputs for downstream tasks through cross-modal alignment. ESIB-Mol^29^ is a deep learning framework that combines the information bottleneck principle with fine-grained substructure experts such as functional groups to filter noise and improve feature extraction capabilities, significantly improving the accuracy and efficiency of molecular property prediction.

## Methods

This section provides a detailed introduction to the proposed framework. For algorithm details, please refer to Section 1 of the Supporting Information.

### 3.1 Simplified Graph Tokenizer

Wu et al.^30^ demonstrated that replacing the nonlinear update function of each GCN layer with a linear transformation in graph GCNs does not significantly affect the performance of GNN models in downstream tasks, but can greatly reduce the complexity of GCNs. As shown as Figure 1A, we proposes a simplified graph tokenizer based on GNNs. By simplifying the nonlinear update function in GNNs and retaining only the core structural relationship information between nodes and edges, the proposed tokenizer avoids overfitting and reduces the computational time and cost during pretraining. The computation of molecular graph representation from input to specific representation can be expressed as follows

1 $$Tok=\left\{y_{i}=\varphi \left({∥}_{k-1}^{K}H_{i}^{\left(k\right)}\right)|i\in V\right\}$$

2 $$H_{i}^{0}=\Phi_{\mathrm{emb}}\left(X\right)$$

3 $$H^{l}=\omega \left(A\right)\cdot H^{l-1}\in R^{\left|V\right|\times d}$$

where Φ_emb_ (·) is the linear layer that shares weights with the encoder in the GAE architecture, and *H*_*i*_^*l*^ represents the feature of the *i*-th node in the *l*-th layer. The initial node features *H*_*i*_^0^ are generated by embedding the node attributes *X* through a linear embedding layer. ω (*A*) denotes the aggregation function based on GNN, which is used to summarize the information on each node’s neighbors, Since Grpah Tokenizer does not participate in training, φ (·) is a stop-gradient operator.

**Figure 1:**
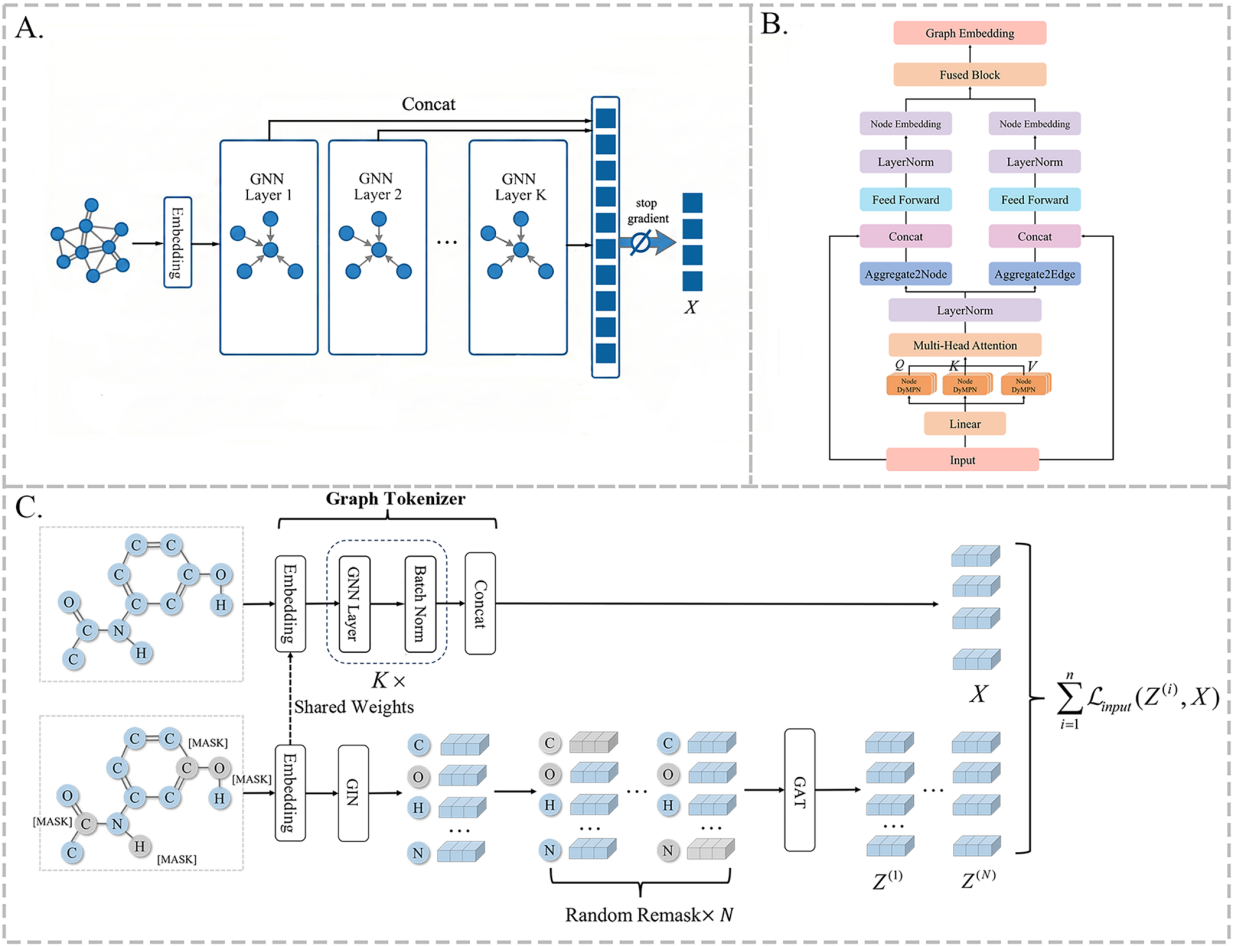
Overview of the TurboGAE model. (A) Framwork of the Graph Tokenizer; (B) illustration of the Graph Transformer in TurboGAE; and (C) framework of the model.

Simplified Graph Tokenizer specializes in feature extraction of root node subtrees, functional groups formed between adjacent nodes, or ring structures. It eliminates the need for complex feature engineering or prior chemical knowledge, making it more widely applicable across a variety of tasks. The proposed tokenizer has no additional trainable parameters, so it can be directly deployed without additional pretraining on large-scale molecular data sets. It can be pretrained alongside TurboGAE, eliminating the pretraining steps required for many graph representation models on large data sets. Tokenizer itself is based on a simplified GNN, simplifying many GNN computational steps while maintaining GNN representation capabilities.

### 3.2 Decoder Based on Graph Transformer

As shown in Figure 1B, we obtain the graph structure representation through a Dynamic Message Passing Network (DMPN) after the graph input, which serves as the input to the multihead attention module. The general message passing process is shown in the following equation

4 $$m_{v}^{l,k}=AGG^{l}\left(\{\left(h_{v}^{l,k-1},h_{u}^{l,k-1},H_{\mathrm{uv}}\right)|u\in N_{v}\}\right)$$

5 $$h_{v}^{l,k}=\sigma \left(W^{l}m_{v}^{l,k}+b^{l}\right)$$

where *m*_*v*_^*l*,*k*^ denotes the aggregated information on node *v*, and *h*_*v*_^*l*,*k*^ is the hidden state of node *v* at the *k*-th hop in the *l*-th layer during the aggregation process. *W* ^*l*^ is the learnable weight matrix used for linear transformation in the *l*-th iteration.

After processing by the multihead attention module and the dynamic message passing module, the hidden states corresponding to nodes *h̅*_*v*_ and edges *h̅*_*vw*_ are obtained, and these states are transformed by the multihead attention module. TheAgg2Node and Agg2Edge modules are used to aggregate the node hidden states to the node embedding and the edge embedding, respectively. The aggregation operations of the Agg2Node and Agg2Edge modules can be represented as follows

6 $$m_{v}^{n2n}=\sum_{u\in N_{v}\;}{\mathrm{h̅}}_{u}$$

7 $$m_{\mathrm{vw}}^{e2n}=\sum_{u\in R_{\mathrm{vw}}\;}{\mathrm{h̅}}_{u}$$

where *N*_*v*_ is the set of neighboring nodes of node *v*, and *R*_*vw*_ is the set of edge (*v*,*w*).

After the Aggregate module, the features of the graph from the node perspective and the edge perspective are obtained. Subsequently, the Graph Transformer employs a long-range residual connection from the input features to the last layer, directly passing the initial node and edge feature information instead of using the multiple short-range residual connections in the original Transformer architecture. The long-range residual connection can alleviate the oversmoothing issue that occurs during message passing, thereby incorporating the information on the entire graph structure into the graph representation. After incorporating the input features, the node embedding are normalized and then enhanced through a feed-forward network to strengthen the feature representation. Finally, after processing, the Graph Transformer module maintains a set of updated node embedding and edge embedding. These embedding are further fused through an MLP module to obtain the final graph feature representation.

### 3.3 Multi-Mask Errors

This section introduces random masks during the decoding process to generate different sets of remasked nodes {*V̅* ^*j*^}_*j*=1,...,*K*_ (where *v*_*i*_∈*V*), with their corresponding hidden feature representations denoted as {*H̅* ^*j*^}_*j*=1,...,*K*_. Each remasked perspective is used to reconstruct the input node features. The randomness in the decoding process acts as a regularizer, preventing the network from learning poorly distinguishable features and thus reducing the sensitivity of training to noise in the input features. Finally, we use the Scaled Cosine Error (SCE) as the loss function for feature reconstruction. The SCE is an improved evaluation criterion for feature reconstruction, specifically designed for graph autoencoder to address the common issues of sensitivity and low selectivity in feature reconstruction. The loss is expressed as follows

8 $$L_{SCE}=\frac{1}{\left|\mathrm{V̅}\right|}\sum_{j=1}^{K}\sum_{x_{i}\;\in V}{\left(1-\frac{x_{i}^{T}z_{i}^{j}}{∥x_{i}∥\cdot ∥z_{i}^{j}∥}\right)}^{\gamma }$$

where γ > 1 is the scaling factor, *X* denotes origin input feature, and *Z* is decoder output.

As shown in Figure 1C, for each batch of input graph data, TurboGAE first performs random masking to generate a masked graph and the mask positions. During the graph representation generation process, Graph Tokenizer is used to extract features from the original input to generate the graph representation. In the forward propagation stage, the masked graph, after being embedded, is processed through a single-layer GCN for feature extraction. Subsequently, multiview mask operations are performed to form different masked feature inputs that are fed into the Graph Transformer-based decoder. After passing through the decoder, different graph representations from various perspectives are obtained. The overall loss is calculated by summing and averaging the SCE losses between the different graph representations and the graph representation obtained by Graph Tokenizer, which serves as the loss for training and fine-tuning. This can be expressed as follows

9 $$L=\sum_{i=1}^{n}L_{SCE}\left(Z_{i},X\right)$$

### 3.4 Multimodal Fusion

After acquiring features of the molecular SMILES sequence and corresponding features of the molecular graph, the SMILES sequence (a one-dimensional textual representation of the molecule) contains information about the order of atoms and chemical bonds within the molecule, and effectively conveys the molecule’s semantic structure. The two-dimensional molecular graph, as a two-dimensional structural diagram, can display the molecule’s topology, atomic connectivity, and chemical properties, providing geometric and topological information. To leverage these two cross-modal features for molecular property prediction, this section proposes three multimodal fusion models.

#### 3.4.1 Dual-Tower Multi-Modal

We designed a feature fusion network based on a dual-tower structure: one network processes molecular graph features, and the other processes SMILES sequence features. The input to the first network is the molecular graph representation processed by the TurboGAE encoder, while the input to the second network is the sequence representation of the SMILES sequence processed by the Transformer. The features based on the molecular graph and SMILES sequence are independently fed into their respective self-attention modules. To enable information interaction between different modalities, cross-attention mechanisms are employed. In this way, the model can not only utilize the features of a single modality but also enhance its adaptability to complex tasks by learning cross-modal relationships. The model structure is shown in Figure 2A.

**Figure 2:**
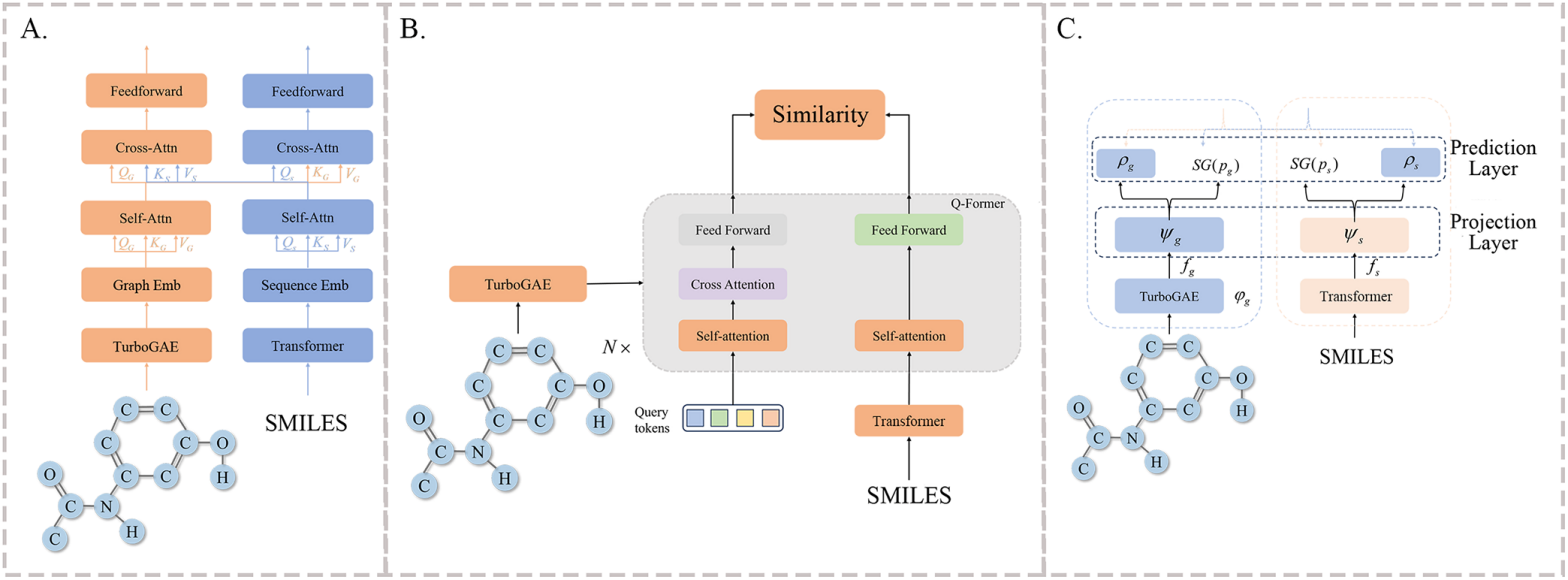
Overview of the multimodal models. (A) Framework based on dual-tower; (B) framework based on Q-Former; and (C) framework based on dual-view consistency.

For the molecular graph representation and SMILES sequence representation of the same molecule, we aim to keep them relatively close in the feature space while maximizing the distance between its molecular graph features and the sequence features of other molecules. For a batch of data samples with batch size *K*, where *x*_1_,...,*x*_*K*_ is the molecular graph representation and *y*_1_,...,*y*_*K*_ is the sequence representation of molecule, the loss function can be described as

10 $$L_{1}=\sum_{i=1}^{K}log\frac{\exp\left(\cos\left(x_{i},y_{i}\right)/\tau \right)}{\sum_{j=1}^{K}exp\left(\cos\left(x_{j},y_{j}\right)/\tau \right)}$$

11 $$L_{2}=\sum_{i=1}^{K}log\frac{\exp\left(\cos\left(y_{i},x_{i}\right)/\tau \right)}{\sum_{j=1}^{K}exp\left(\cos\left(y_{j},x_{j}\right)/\tau \right)}$$

12 $$L=-\frac{1}{K}L_{1}-\frac{1}{K}L_{2}$$

where τ is the temperature coefficient, which is mainly used to adjust the scale of the similarity to avoid overly extreme similarity values.

#### 3.4.2 Q-Former Multi-Modal

As shown in Figure 2B, we achieve alignment between molecular sequence modalities and molecular graph modalities based on the Q-Former architecture,—a lightweight transformer structure. The disadvantage of the dual-tower architecture is that its two independent encoding processes may require more adjustments and optimizations during the feature fusion stage to ensure effective alignment between the different modalities. However, the Q-Former directly fuses features from different modalities in the self-attention and cross-attention layers, enabling alignment of information from both modalities at a deeper level. Similar to the loss function of the dual-tower structure, the loss function can be described as

13 $$L_{1}=\sum_{i=1}^{K}log\frac{\exp\left(max_{k}c\mathrm{os}\left(m_{\mathrm{ik}},t_{i}\right)/\tau \right)}{\sum_{j=1}^{K}exp\left(max_{k}\cos\left(m_{\mathrm{ik}},t_{j}\right)/\tau \right)}$$

14 $$L_{2}=\sum_{i=1}^{K}l\mathrm{og}\frac{\exp\left(\mathrm{ma}x_{k}\cos\left(t_{i},m_{i}\right)/\tau \right)}{\sum_{j=1}^{K}e\mathrm{xp}\left(\mathrm{ma}x_{k}\cos\left(t_{i},m_{\mathrm{jk}}\right)/\tau \right)}$$

15 $$L=-\frac{1}{K}L_{1}-\frac{1}{K}L_{2}$$

where *m*_*ik*_ is representation of molecular graph, *t*_*i*_ is representation of SMILES sequence. The setting of τ is consistent with the loss in the dual-tower structure.

#### 3.4.3 Dual-View Consistency Multi-Modal

In the traditional dual-tower structure, molecular graph features and sequence features are usually processed independently by their respective models. Subsequently, cross-attention mechanisms are employed to facilitate interaction and alignment between these two types of features. The limitation of this approach is that the dual-tower structure itself does not consider directly optimizing the consistency between different modalities during the pretraining stage. Although alignment between different modalities can be promoted through contrastive loss or cross-attention mechanisms in the later stages, this alignment is indirect and mainly focused on the fusion layer. This approach may lead to insufficient constraints on the intermodal differences in the feature space, thereby affecting the model’s performance in practical applications, especially in complex tasks where the representations of the two modalities may not form a completely consistent semantic space.

We aim to maximize the representation consistency between molecular graph features and sequence features for the same molecule as the training objective of the model. This can be achieved by designing a novel loss function, which aims to minimize the differences between the molecular graph features and sequence features of the same molecule or molecules with similar chemical structures. This approach promotes tight alignment between the two modalities without relying on negative samples.

Figure 2C illustrates the overall framework of the multimodal fusion strategy based on dual-view consistency. The encoder of TurboGAE is used as the encoding module for molecular graphs, and transformer is employed to represent the features of SMILES sequences. The projection layer consists of a 3-layer MLP network to enhance the model’s nonlinear expression capability and stability. The prediction layer is composed of a 2-layer MLP network. The overall representation process can be expressed as follows

16 $$p_{g}=\psi_{g}\left(f_{g}\right),q_{g}=\rho_{g}\left(p_{g}\right)$$

17 $$p_{s}=\psi_{s}\left(f_{s}\right),q_{s}=\rho_{s}\left(p_{s}\right)$$

where ψ_*g*_ and ψ_*s*_ are projection layers, ρ_*g*_ and ρ_*s*_ are prediction layers. The dual-view consistency loss is defined as

18 $$L_{\mathrm{dual}}=\left({\mathrm{M̃}}_{g},{\mathrm{M̃}}_{s};\varphi_{g},\varphi_{s},\psi_{g},\psi_{s},\rho_{g},\rho_{s}\right)=-\cos\left(q_{s},\mathrm{SG}\left(p_{g}\right)\right)-\cos\left(q_{g},\mathrm{SG}\left(p_{s}\right)\right)$$

where *M̃*_*g*_ and *M̃*_*s*_ are the masked molecular graph and sequence representations, respectively, and SG (·) is the stop-gradient operator.

To align the dimensions of the TurboGAE molecular map and the SMILES sequence, we employed specific alignment strategies for each fusion strategy. For Dual-Tower and Dual-view Consistency, we utilize independent Multi-Layer Perceptrons (MLPs) as projection heads. Each MLP consists of a Linear layer, a ReLU activation and a final Linear layer. This projects both graph and sequence representations into a latent space of the same dimension. And for Q-Former, the dimension alignment is handled implicitly via the cross-attention mechanism. The learnable query vectors interact with the encoder outputs through attention projection matrices that naturally accommodate the differing input dimensions of the graph and sequence encoders.

## Results

### 4.1 Data Sets and Preprocessing

The ZINC database^31^ is currently the largest database of organic small molecule structures, containing over 980 million small molecules. The PubChem database^32^ is currently the largest global repository of publicly available chemical molecules and related information, including structural data of millions of chemical molecules, such as small molecules, organic compounds, inorganic compounds, and mixtures. MoleculeNet^33^ is an open benchmark platform focused on molecular property prediction, and it integrates a variety of chemical and biochemical characterization methods. The downstream subdata sets mainly used in the experimental part of this paper include: BBBP, Tox21, ToxCast, Sider, ClinTox, MUV, HIV, Bace, ESOL, FreeSolv, Lipophilicity. For more information, please see Table 1.

**Table 1:** Statistics of Datasets

data set	task type	#tasks	#compounds	split	metrics
BBBP	classification	1	2039	scaffold	ROC-AUC
Tox21	classification	12	7831	scaffold	ROC-AUC
ToxCast	classification	617	8576	scaffold	ROC-AUC
SIDER	classification	27	1427	scaffold	ROC-AUC
ClinTox	classification	2	1477	scaffold	ROC-AUC
MUV	classification	17	93,087	scaffold	ROC-AUC
HIV	classification	1	41,127	scaffold	ROC-AUC
BACE	classification	1	1513	scaffold	ROC-AUC
ESOL	regression	1	1128	random	RMSE
FreeSolv	regression	1	642	random	RMSE
lipophilicity	regression	1	4200	random	RMSE

For pretraining and fine-tuning, 2,500,000 molecular sequences were randomly sampled from two large molecular databases (ZINC and PubChem), with the SMILES sequence length restricted to 125 characters. The selection of a 125-character limit is based on the statistical distribution of drug-like chemical space. Analysis of the ZINC and PubChem databases indicates that the vast majority (>95%) of pharmacologically relevant small molecules (typically MW < 500 Da) possess Canonical SMILES strings significantly shorter than 125 characters, with a mean length often between 30 and 60 characters. Molecules exceeding this length are typically large macrocycles, polymers, or biomolecules that fall outside the applicability domain of standard small-molecule property prediction tasks found in MoleculeNet. Furthermore, limiting sequence length is a standard practice to manage the computational complexity of Transformer-based attention mechanisms without sacrificing coverage of relevant chemical space.

We chose scaffold splitting over random splitting because it enforces a stricter test of generalization. It ensures that the test set contains molecules with structural backbones different from those in the training set, thereby simulating the real-world drug discovery scenario of out-of-distribution generalization.

### 4.2 Main Results

This experiment mainly compares TurboGAE with the following baseline models: Infomax,^34^ MPNN,^35^ GraphMAE, GraphMAE2, MGSSL,^36^ GraphCL, GraphMVP, GROVER, MOLE-BERT, LGGA and ESIB-Mol. And using ROC-AUC as the evaluation metric. AUC is an important indicator for assessing the performance of classification models. It reflects the overall performance of the model under different decision thresholds and can measure the model’s ability to distinguish between positive and negative samples. For the settings of training parameters, please refer to Supporting Information Section 2.

As shown in Figure 3, the classification tasks’ results show that TurboGAE achieved the highest AUC on the majority of MoleculeNet subsets, significantly outperforming the contrastive learning methods InfoMax and GraphCL, which validates the effectiveness of the GAE architecture for 2D graph representation. Although GraphMVP which incorporates 3D information, performed slightly better on Tox21, its pretraining cost is prohibitively high. The generative pretraining methods MOLE-BERT and GROVER also led in specific tasks but rely on complex pretraining strategies. Overall, TurboGAE achieved the best performance on multiple subdata sets. Its representation of 2D molecular graph structures is more comprehensive compared to other molecular graph models. Moreover, TurboGAE significantly reduces the training cost of training graph tokenizers on data sets, demonstrating its effectiveness in molecular property prediction tasks.

**Figure 3:**
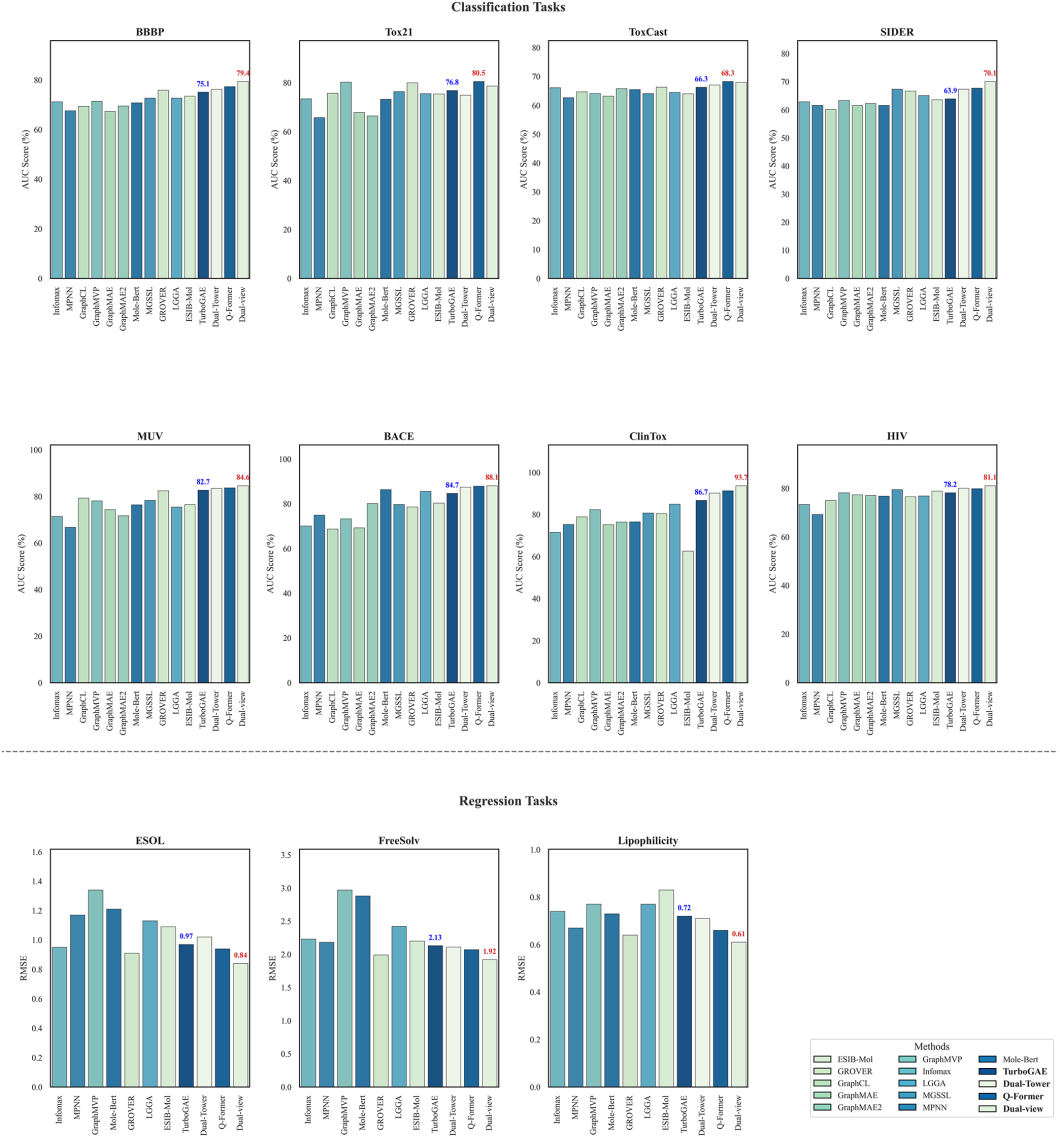
Performance of our models on classification and regression data sets. We conducted the experiment using 5 random seeds, and the result is the mean. We performed a paired *t* test to verify the significance of our improvements. The results show that our model significantly outperforms the baseline (*p* = 0.032).

We then verified the performance of the fusion strategies based on the dual-tower model, Q-Former, and dual-view consistency, as well as TurboGAE. The experimental comparison results are shown in the lower part of Figure 3. The multimodal fusion strategies based on dual-view consistency outperforms the dual-tower structure and Q-Former. The test data sets contain a large number of molecules from completely different chemical spaces, exhibiting strong data heterogeneity. The dual-tower structure processes the features of each modality relatively independently, which limits its performance in handling heterogeneous data. In contrast, the strategy based on dual-view consistency optimizes both the graph representation and the sequence representation of molecules simultaneously, enabling it to better capture the differences in heterogeneous data. Overall, the dual-tower structure can reduce the interference of mutual information between modalities but lacks the sharing of common information across different modalities. The strategy based on Q-Former goes deeper in feature fusion than the dual-tower structure but is less flexible. The strategy based on dual-view consistency can enforce feature alignment between modalities, enhancing representation ability and is suitable for complex tasks, but it incurs a significant additional computational cost.

In the regression tasks, where a lower RMSE indicates better performance, our models maintained their lead. Dual-view achieved the lowest error rates on all three data sets. Interestingly, the base TurboGAE model proved highly effective for regression, achieving the second-lowest RMSE on ESOL (0.93) and FreeSolv (2.13), outperforming complex baselines like GROVER and GraphMAE2. Overall, these results validate the effectiveness of our proposed architecture in learning expressive molecular representations.

### 4.3 Ablation Studies and Parameter Sensitivity Analysis of TurboGAE

In this section, we analyze the components and parameters of TurboGAE. Unless otherwise specified, we conducted experiments on data sets for eight classification tasks and took the average value. For TurboGAE, when the encoder is set to graph attention network (GAT),^37^ graph isomorphism network (GIN),^38^ and GCN, and the decoder is set to a linear layer, Figure 4A shows that the combinbation of GIN-based encoder and Graph Transformer is the most suitable model structure. GIN captures the relationships between nodes in the graph better than traditional GNNs or GCNs. For data sets with a large number of similar subgraphs, such as functional groups or ring structures, GIN can better distinguish the isomorphism of graphs. Moreover, as the number of network layers increases, the node features of the graph structure tend to become more similar. GIN can effectively avoid over-reliance on neighboring nodes. Figure 4B show the change in average loss during the pretraining stage as the number of epochs increases. In the pretraining stage, the model’s loss is highly related to the complexity of the Decoder. Using a linear network structure or a Decoder architecture based on GNN results in significantly higher training loss compared to using a Decoder architecture based on Graph Transformer. This indicates that the choice of Decoder plays a key role in graph-structured molecular representation learning. A more complex Decoder structure can better capture long-range dependencies and complex structural patterns in the graph, thereby improving feature learning and reconstruction capabilities.

**Figure 4:**
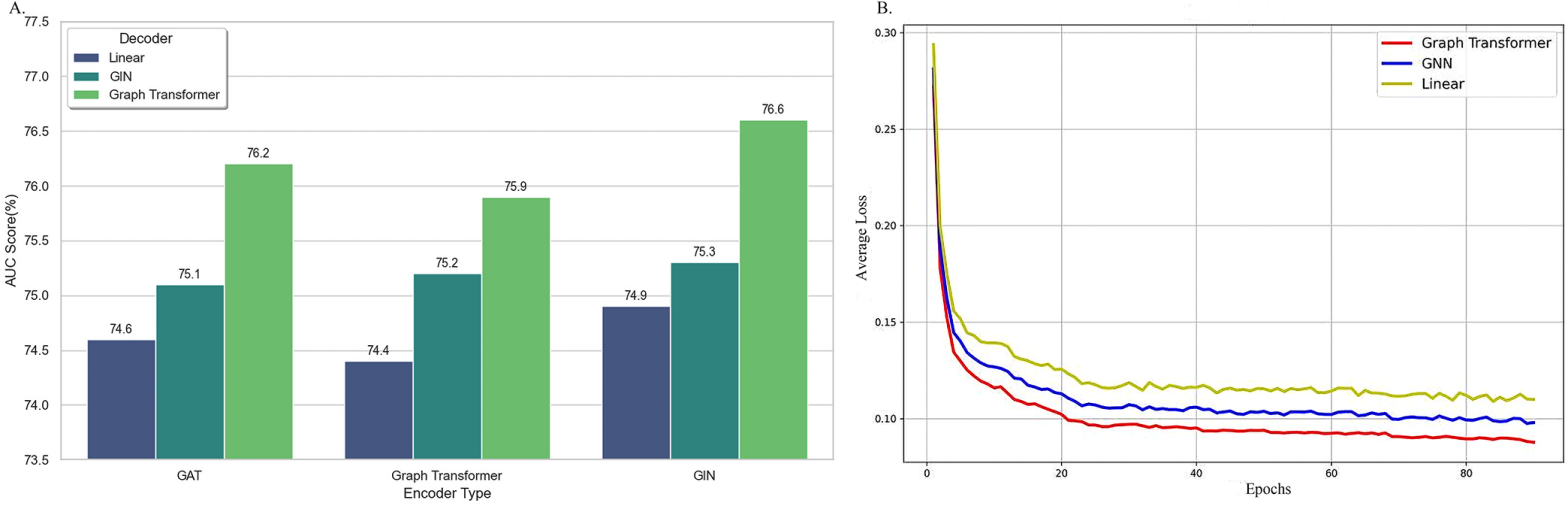
Performance of encoders and decoders with different structures. (A) Results of different decoder and encoder networks. (B) Impact of decoder architecture on model performance.

Regarding the multiple mask error strategies proposed earlier, experiments were conducted with strategies such as no remask, single remask, and multiview mask. No Remask represents the standard Autoencoder approach. The encoder processes a masked graph, and the decoder attempts to reconstruct the original features directly from the encoder’s latent output. Single remask replaces the masked node features with another mask token before they are input into the decoder. Multiview mask generates independent remasked views of the latent representation. The model must simultaneously optimize the reconstruction loss across all views. This acts as a powerful consistency regularizer, reducing the variance of the gradient estimates and preventing overfitting to noise. The overall performance comparison is shown in Figure 5A. Mask strategies alter the complexity of the network structure, and model performance increases with the complexity of the network structure. This is because the decoder’s expressive power may be too weak, for example, using a linear decoder, which may not be sufficient in extracting and representing data features, thus not adequately supporting downstream tasks for transfer learning or fine-tuning. More complex decoder structures, based on feature reconstruction, can enhance the universality and representation ability of the encoding. Combining remask strategies not only enhances the decoder’s reconstruction ability but also prevents the network from learning too many features with poor distinguishability.

**Figure 5:**
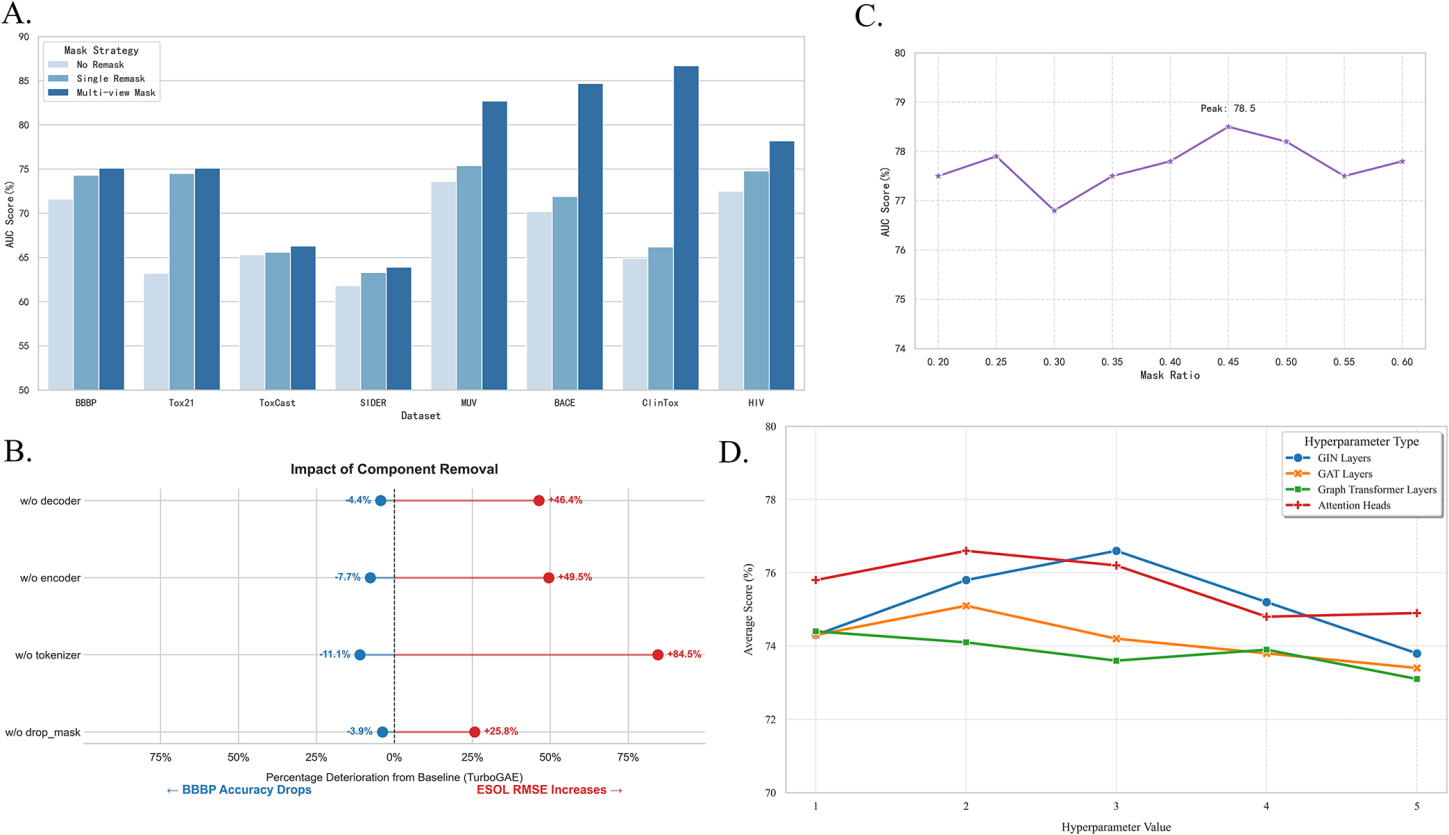
(A) Performance of different mask strategies. (B) Ablation Studies of TurboGAE. (C) Task performance curve with mask ratio. (D) The impact of hyperparameters on results.

To investigate the impact of key components of TurboGAE on the results, we conducted ablation experiments on the classification task BBBP and the regression task ESOL. The results are shown in Figure 4B. After removing the Tokenizer, the AUC of BBBP decreased by 11.1%, while the RMSE of ESOL increased by 84.5%. This significant decrease in results demonstrates the importance of the Graph Tokenizer as a core component. Second, removing the Encoder resulted in a 7.7% decrease in the AUC of BBBP and a 49.5% increase in the RMSE of ESOL, making it second only to the Tokenizer in importance. All data points in the figure show performance degradation, indicating that the TurboGAE model is a compact system without unnecessary components. Furthermore, the ESOL task is extremely sensitive to missing components, with a very large increase in error, while the BBBP task, although also affected, experiences relatively smaller fluctuations. This may suggest that regression tasks rely more heavily on the fine structure of the model than classification tasks.

The impact of the mask ratio on the model was also verified. Since multiple mask errors were introduced before the input to the decoder, an excessively high mask ratio may lead to the decoder’s inability to effectively reconstruct node features. The results is shown in Figure 4C that for TurboGAE, the overall model performance is best when the mask ratio is 0.45. Starting from a mask ratio of 0.3, the overall performance first increases and then decreases with the increase of the mask ratio. This is because at a lower mask ratio, the Graph Transformer cannot fully utilize its advantages to complete feature reconstruction, while an appropriate mask ratio can fully leverage the decoder to complete the feature reconstruction task.

We designed a sensitivity experiment on hyperparameters to verify the impact of different parameter sizes on TurboGAE. The results are shown in Figure 5D. The model performs best when the number of attention heads is 2 and the number of GIN layers is 3. The GAT model seems to be more suitable for shallow networks; deep GAT does not extract more effective information and instead introduces noise or leads to overfitting. Graph Transformer performs well as a decoder, but it has no advantage as an encoder and is far inferior to GIN and GAT.

To further demonstrate TurboGAE’s generalization ability, we conducted experiments on a small data set following the methodology outlined in TransFoxMol. For detailed information on the data set, please refer to Section 3 of the Support Information. As shown in Figure 6A, TurboGAS exhibits significant performance variations across different data sets. For instance, on CHEMBL3215081 and CHEMBL900190, the model demonstrates high ROC-AUC and PRC-AUC. The results for the CHEMBL1613998 data set show shorter bins, indicating that the results from multiple experiments are relatively concentrated and the performance is quite stable. Overall, while TurboGAE’s performance declines on smaller data sets, it does not show a significant difference compared to larger data sets, demonstrating its good generalization ability on smaller data sets.

**Figure 6:**
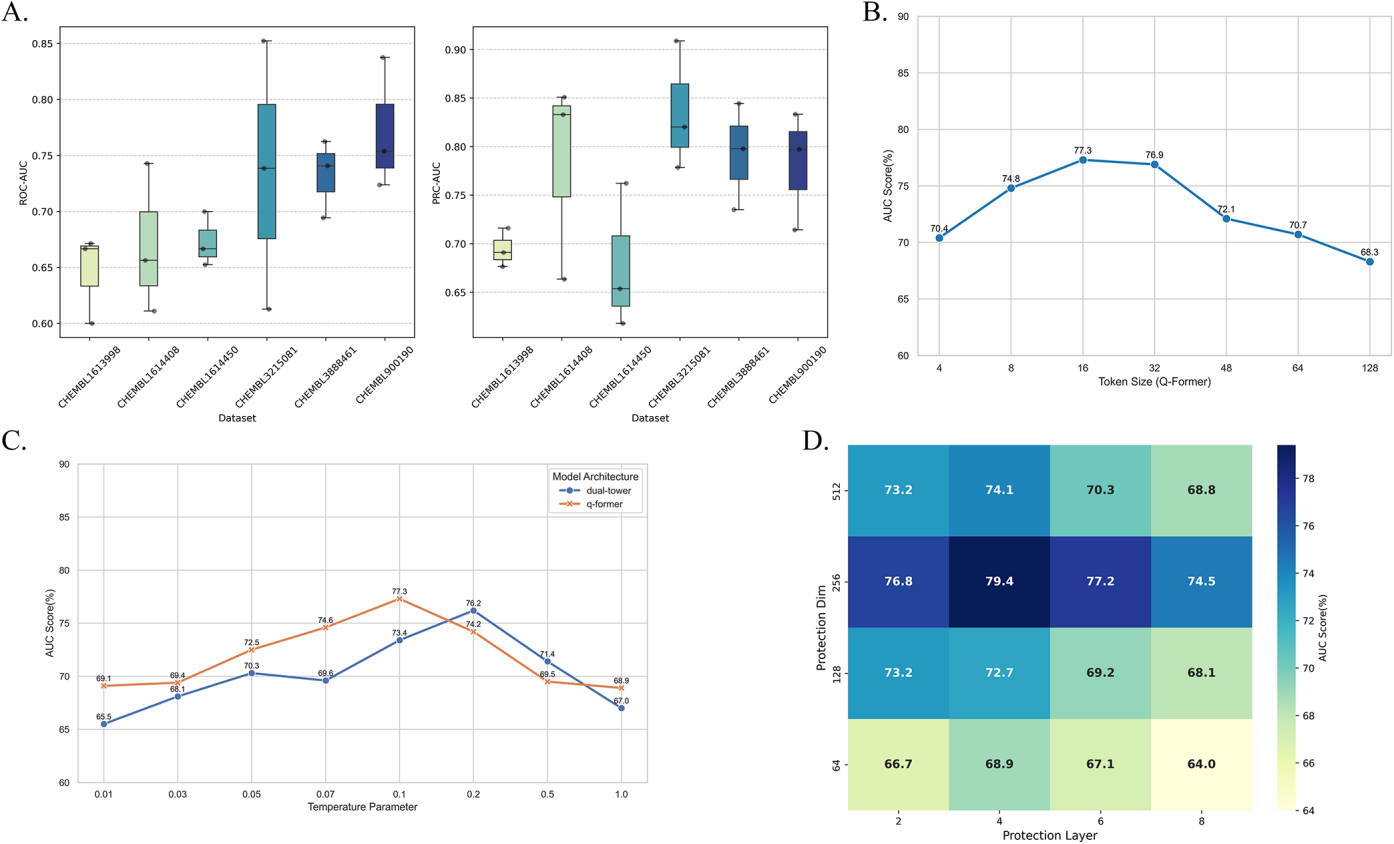
(A) TurboGAE’s performance on small data sets. (B) The effect of token size on q-former. (C) The effect of temperature coefficient on dual-tower and q-former. (D) The performance of the dual-view projection layer under different parameter combinations.

### 4.4 Ablation Studies of Multi-Modal Models

Our proposed multimodal model performs well. To explore the impact of parameter variations in different models, we conducted a comparative experiment on the parameters of the multimodal model. Figure 6B illustrates the relationship between Q-Former’s token size and AUC score, with optimal performance achieved when the token size is 16. The number of tokens in Q-Former determines the amount of information extracted from the original molecular features. With only 4 or 8 tokens, the information window is too narrow, making it impossible to fully capture the key fingerprint information of the molecular structure. Too many tokens, however, do not bring more effective information but instead introduce noise. In the attention mechanism, too many queries can distract the attention weights, causing the model to focus on irrelevant features, thus reducing its discriminative ability.

Figure 6C illustrates the impact of the key Temperature parameter on the model architecture. Q-Former outperforms Dual-tower under almost all parameters, demonstrating that this cross-attention-based interaction captures more complex molecular features than simple dot products. Temperature controls the smoothness of the Softmax distribution, so smaller parameters result in more effective discrimination of negative samples. As shown in Figure 6C, Q-Former is more sensitive to Temperature, achieving optimal performance at 0.1, while Dual-tower requires a larger Temperature to reach its optimal state. This reflects that to obtain the same feature information, the dual-tower structure requires a larger representation space.

The projection layer is a crucial component of the dual-view model, enabling feature alignment between the SMILES sequence and the molecular map. Figure 6D illustrates the differences in the combination of the number and dimensionality of the projection layers. While increasing the number of projection layers from 2 to 4 layers improves performance, the effect significantly deteriorates after 4 layers, suggesting vanishing gradients or overpropagation of features leading to information loss. The projection layer’s dimensionality is biased toward higher dimensions, reaching its optimum at 512, indicating that low-dimensional spaces cannot adequately represent complex molecular features. The best-performing combination is four 256-dimensional projection layers. As can be seen from Figure 6D, the combination of high-dimensional space and lower layer count performs well overall, but the combination of low-dimensional space and high-dimensional space performs poorly, and features are continuously compressed and eventually lost during propagation in deep networks.

### 4.5 Interpretability Analysis

Figure 7A illustrates the contribution of different atom types to the model’s predictions. Nitrogen has the highest average importance, followed by carbon and sulfur. This aligns well with the intuition of medicinal chemistry, as nitrogen atoms are typically hydrogen bond donors or acceptors, serving as key sites for the interaction between drug molecules and biological targets.^39^ It can also be seen that the standard deviations for nitrogen, carbon, sulfur, and oxygen are very large, indicating that the importance of these atoms fluctuates significantly across different molecules. Nitrogen atoms may be active sites in some molecules, while less important in others. The standard deviations for other elements are smaller, suggesting that the contributions of halogen and hydrogen atoms are relatively stable. In summary, the model relies primarily on the nitrogen, carbon, and sulfur scaffolds and their polar characteristics rather than solely on halogen substituents for predictions.

**Figure 7:**
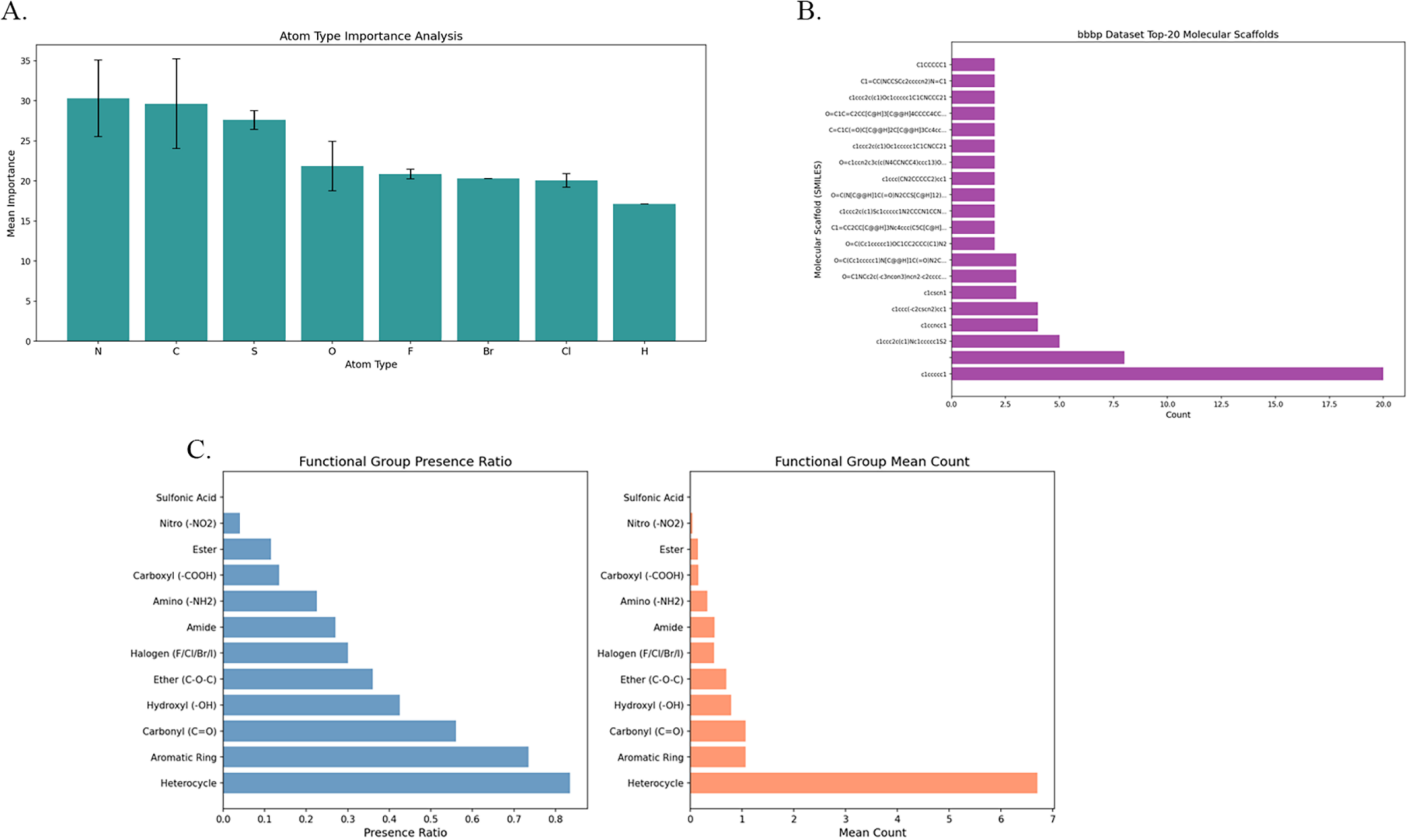
Results of interpretability analysis. (A) Result of atoms importance analysis. (B) Top-20 molecular scaffolds on bbbp data set. (C) Result of functional group.

Figure 7B shows the 20 most frequently predicted molecular scaffolds in the Blood-Brain Barrier Permeability Data set (BBBP). The benzene ring ranks first, being the most common structural unit in medicinal chemistry. The second most frequent skeleton has a count of around 8, and most subsequent scaffolds have counts between 2 and 5. Therefore, even the top 20 scaffolds are relatively few in number compared to the BBBP data set. This suggests that no single complex scaffolds dominates the data set, and permeability may depend more on side chains or other molecular properties.

In the BBBP data set, a high proportion of heterocyclic and aromatic rings typically helps modulate the lipophilicity of molecules, a key factor in penetrating the blood-brain barrier. As shown on the left side of Figure 7C, most molecules contain heterocyclic and aromatic rings. This is typical of drug-like molecules because ring structures provide a rigid framework, and heterocyclic rings modulate the physicochemical properties of molecules.^40^ The right side of Figure 7C shows a high average number of heterocyclic rings, meaning that a single molecule may contain multiple heterocyclic rings. This also illustrates the high complexity of molecular structures in the BBBP data set.

## Conclusion

Drug analysis and design is an important research direction in cutting-edge disciplines. Molecules, as the basic components of drugs, have properties that are of significant reference value for related applications. Currently, deep learning has been widely applied to molecular property prediction tasks. However, most previous deep learning-based methods have adopted single-modal molecular representations and have been inadequate in representing different modalities. There is still considerable room for improvement in the complexity of models and strategies.

To address the above issues, we have conducted research on the representation of different modalities of molecules and the fusion of features between different modalities. We propose TurboGAE, an efficient graph autoencoder, for large-scale graph data, as well as a multimodal fusion framework. TurboGAE significantly reduces the training cost of tokenizers for large-scale graphs through a subgraph-level graph tokenizer. The decoder is replaced with an attention-based complex GNN and incorporates multiview masking and error suppression mechanisms, which greatly improves reconstruction accuracy. Finally, the framework integrates three complementary fusion strategies: dual-tower, Q-Former, and dual-view consistency, to achieve robust multimodal alignment. It has shown excellent performance on various data sets. In the context of high-throughput virtual screening (HTVS), even a 1–2% improvement in ROC-AUC can translate to a significant increase in the Enrichment Factor (EF). This means that when screening a library of millions of compounds, the top-ranked fraction will contain significantly more true actives and fewer false positives. This directly translates to cost savings in the wet lab, as fewer compounds need to be synthesized and tested to find a lead candidate. TurboGAE’s contribution is thus not just statistical but economically relevant for drug development pipelines.

Our experiments mainly focus on downstream task prediction based on molecular SMILES sequences and two-dimensional molecular graphs. However, the input molecular graphs are based solely on the topological structures of molecules and do not incorporate relevant spatial information—also crucial for molecular properties. Future work can explore the use of different modalities of representation and the introduction of more features from other modalities to improve the model.

## Supplementary Material



## Data Availability

The benchmark data sets are sourced from MoleculeNet (https://moleculenet.org/datasets-1). The code for model execution has been released on GitHub (https://github.com/kytieguo/Multi-ViewSGT).

## Notes

Algorithms; parameter settings; details of small data sets (PDF)
