# Supplementary material for: Multi-Modal Fusion Frameworks of Subgraph-Optimized Graph Autoencoder for Molecular Property Prediction
Source: J Chem Inf Model. 2026 Jan 28;66(3):1789–800. doi: 10.1021/acs.jcim.5c02536 (PMC12892327; doi:10.1021/acs.jcim.5c02536)
Supplement: Supplementary file 1 [file ci5c02536_si_001.pdf]

Multi-Modal Fusion Frameworks of Subgraph-Optimized Graph Autoencoder for Molecular Property Prediction

Kaiyuan Zhang[a], Congyu Han[a], Fenghua Zhang[b], Cheng Lin[b], Quanlong Li[a], Tianyi Zang[a*], and Yanli Zhao[c*]

[a] *Faculty of Computing, Harbin Institute of Technology, Harbin, Heilongjiang 150001, China.*

[b] *National key laboratory of science and technology on advanced composites in special environments, Harbin Institute of Technology, Harbin, Heilongjiang 150001, China.*

[c] *Medical College, Qinghai University, Xining, Qinghai 810016, China.*

* Email: tianyi.zang@hit.edu.cn

* Email: yanli_2000@163.com

1. ALGORITHMS

The following are the algorithms of the models proposed in the main documentary, including TurboGAE and multi-modal fusion models.

Table S1. Algorithm of TurboGAE

Algorithm 1: TurboGAE

Input: Molecular graph g, gnn layers of tokenizer n, random masking times N.
Output: Molecular property prediction labels
repeat:
for g **in** loader **do**:
  For molecular graphs, enter a random mask: g, mask_position←random_mask (g)
  Get information about atoms and bonds: x, edge_index←g
  for $i = 1$ **to** $n - 1$ **do**:
    Message passing: x←propagate(embed(x), edge_index)+(1+eps)*embed(x)
    Normalization: x←batchnorm(x)
  end
  Decoder: $\widehat{z_i}$←GAT(remask(GCN(x)), mask_position)
  for $i = 1$ **to** $N - 1$ **do**:
    Calculate Scaled Cosine Error: loss←$\sum_{i=1}^{N} \mathcal{L}_{\text{input}} (z_i[mask\_position], X)$
  end
end
until: Model convergence
labels←Classify(TurboGAE(g))
return

Table S2. Algorithm of Dual-Tower Multi-Modal

Algorithm 2: Dual-Tower Multi-Modal

Input: Molecular graph g, Molecular SMILES x.
Output: Representation after molecular features fusion Z
Function: Dual_Tower(g, x)
    f_{g_rep}←TurboGAE(g)
    f_{s_rep}←Transformer(x)
    f_g, f_s←Cross_Attention(Self_Attention(f_{g_rep}), Self_Attention(f_{s_rep}))
    Z←Feed_Forward(f_g), Feed_Forward(f_s)
    return Z
Z←Dual_Tower(g, x)
return Z

Table S3. Algorithm of Q-Former Multi-Modal

Algorithm 3: Q-Former Multi-Modal

Input: Molecular graph g, Molecular SMILES x.
Output: Representation after molecular features fusion Z
Function: Q_Former(H_Graph, H_Sequence)
Q←Init(H_Graph)
Q_{Graph}←Self_Attention(Q, H_Graph)
Q_{Seq}←Cross_Attention(Q_{Graph}, H_Sequence)
Q_{total}←Self_Attention(Q_{Graph}, Q_{Seq})
Z←Feed_Forward(Q_{total})
return Z
H_Graph←TurboGAE(g)
H_Sequence←Transformer(x)
Z←Q_Former(H_Graph, H_Sequence)
return Z

Table S4. Algorithm of Dual-view Consistency Multi-Modal

Algorithm 4: Dual-view Consistency Multi-Modal

Input: Molecular graph g, Molecular SMILES x.
Output: Molecular property prediction labels
repeat:
for x, g **in** loader **do**:
Get molecular graph representation via TurboGAE: f_g←TurboGAE(g)
Get molecular sequence via Transformer: f_s←Transformer(x)
Projection layer: $p_g \leftarrow \psi_g(f_g)$, $q_g \leftarrow \rho_g(p_g)$; $p_s \leftarrow \psi_s(f_s)$, $q_s \leftarrow \rho_s(p_s)$
Calculate loss：$\text{loss} \leftarrow -\cos(q_s, SG(p_g)) - \cos(q_g, SG(p_s))$
end
until: Model convergence
Prediction function:
f_g←TurboGAE(g)
f_s←Transformer(x)
f_{fusion}←DeepFuse(f_s, f_g)
labels←Classify(f_{fusion})
return labels

2. PARAMETERS SETTING

Table S5. TurboGAE parameter settings

Parameters	Settings
gnn_emb_dim	300
gnn_token_layer	1
gnn_encoder_layer	1
num_attn_head	2
batch_size	256
mask_ratio	0.45
epochs	100
decoder_attn_head	3
learning_rate	1e-5

Table S6. Dual-tower parameter settings

Parameters	Settings
n_layers	4
latent_dim	512
num_heads	12
τ	0.2
optimizer	Adam
dropout	0.1
batch size	256
epochs	30
learning rate	5e-5

Table S7. Q_Former parameter settings

Parameters	Settings
n_query	16
latent_dim	512
n_layers	6
num_heads	12
prediction_layers	2
τ	0.1
optimizer	Adam
dropout	0.1
batch size	256
epochs	30
learning rate	5e-5

Table S8. Dual-view Consistency parameter settings

Parameters	Settings
projection_dim	256
projection_layers	4
hidden_dim	256
prediction_dim	128
prediction_layers	2
optimizer	Adam
dropout	0.1
batch size	256
mask_ratio	0.3
epochs	30
learning rate	1e-4

3. DETAILS OF SMALL DATASETS

Table S9. Details of small-scale datasets.

ChEMBL ID	Target name	Molecules	Positive rate(%)
1613998	Janus Kinase 2(JAK2)	101	51.5
1614408	Nuclear Receptor subfamily 2 group E member 3(NR2E3)	114	50.0
1614450	Aryl Hydrocarbon Receptor(AHR)	124	50.0
3215081	Non-Protein target	182	50.0
3888461	Tankyrase 1(TANK)	173	49.7
900190	Transthyretin(TTR)	92	50.0
